# Are standardized caries risk assessment models effective in assessing actual caries status and future caries increment? A systematic review

**DOI:** 10.1186/s12903-018-0585-4

**Published:** 2018-07-16

**Authors:** Maria Grazia Cagetti, Giuliana Bontà, Fabio Cocco, Peter Lingstrom, Laura Strohmenger, Guglielmo Campus

**Affiliations:** 1 Department of Biomedical, Surgical and Dental Sciences, University of Milan, Via Beldiletto 1, 20142 Milan, Italy; 2 WHO Collaboration Centre for Epidemiology and Community Dentistry, Via Beldiletto 1, 20142 Milan, Italy; 3 Department of Surgery, Microsurgery and Medicine Sciences, School of Dentistry University of Sassari, Viale San Pietro, 43 Sassari, Italy; 4 Department of Cariology, Institute of Odontology, The Sahlgrenska Academy, University of Gothenburg, Medicinaregatan 12 A-G, P.O. Box 450, 405 30 Gothenburg, Sweden

**Keywords:** Dental caries, Dental caries susceptibility, Dental health surveys, Risk assessment, Review

## Abstract

**Background:**

Assessing caries risk is an essential element in the planning of preventive and therapeutic strategies. Different caries risk assessment (CRA) models have been proposed for the identification of individuals running a risk of future caries. This systematic review was designed to evaluate whether standardized caries risk assessment (CRA) models are able to evaluate the risk according to the actual caries status and/or the future caries increment.

**Methods:**

Randomized clinical trials, cross-sectional studies, cohort studies, comparative studies, validation studies and evaluation studies, reporting caries risk assessment using standardized models (Cariogram, CAMBRA, PreViser, NUS-CRA and CAT) in patients of any age related to caries data recorded by DMFT/S or ICDAS indices, were included. PubMed, Scopus and Embase were searched from 2000 to 2016. A search string was developed. All the papers meeting the inclusion criteria were subjected to a quality assessment.

**Results:**

One thousand three-undred ninety-two papers were identified and 32 were included. In all but one, the Cariogram was used both as sole model or in conjunction with other models. All the papers on children (*n* = 16) and adults (*n* = 12) found a statistically significant association between the risk levels and the actual caries status and/or the future caries increment. Nineteen papers, all using the Cariogram except one, were classified as being of good quality. Three of four papers comprising children and adults found a positive association. For seven of the included papers, Cariogram sensibility and specificity were calculated; sensibility ranged from low (41.0) to fairly low (75.0), while specificity was higher, ranging from 65.8 to 88.0. Wide 95% confidence intervals for both parameters were found, indicating that the reliability of the model differed in different caries risk levels.

**Conclusions:**

The scientific evidence relating to standardized CRA models is still limited; even if Cariogram was tested in children and adults in few studies of good quality, no sufficient evidence is available to affirm the method is effective in caries assessment and prediction. New options of diagnosis, prognosis and therapy are now available to dentists but the validity of standardized CRA models still remains limited.

**Electronic supplementary material:**

The online version of this article (10.1186/s12903-018-0585-4) contains supplementary material, which is available to authorized users.

## Background

Different caries risk assessment (CRA) models have been proposed for the identification of individuals running a risk of future caries [1–7].

Caries is a multifactorial disease resulting from a series of events occurring in a chain that lasts for years where clinical, microbiological, behavioral and social factors are involved in the process. In view of its multifactorial nature, a multivariate approach is necessary [8]. The scientific basis for caries risk assessment, prevention and treatment on an individual patient basis requires incessant development, specification and continuing validation [9]. Scientific evidence proving CRA methods’ validity is limited [3]. Past caries experience is regarded as the single most powerful caries predictor in all age groups [4–7, 10]. Different measures of past caries experience are often included in analytical models of multi-risk studies. Nevertheless, there are consequences of including past caries experience measures for both prediction and multi-risk models since this parameter will hide the effects of weaker indicators of high risk individuals or of other caries risk-factors [11].

Caries risk assessment still has great potential to enhance patient care as it is the corner stone of a minimal invasive care plan, allowing the determination of the appropriate non-invasive as well as invasive interventions and recall strategies [12], but still today, a great need to standardize study design, outcome measures and reporting of data in studies on CRA is required [13].

Standardized models including different combinations of risk and protective factors (Table 1) have been developed from the 2000s onwards to predict caries; they can be summarized in two main categories, those using an algorithm with a software program and those using standardized questionnaires (self-submitted and/or through an interview). Moreover, CRA methods could be used as an effective health-education tool to change the attitudes and behaviors of patients/parents/caregivers towards good oral hygiene and dietary habits maintenance [14].

**Table 1:** Different factors included in each standardized Caries Risk Model

	Software programs			American Dental Association models		
Factors	NUS-CRA 11 factors	Cariogram 9 factors	PreViser 11 factors	ADA 11 factors	CAMBRA 14 factors	CAT 12 factors
*Socio-demographic*						
Age	X		X			
Ethnicity	X					
Family socioeconomic status	X			X	X	X
*Behavioural*						
Infant feeding history	X				X	
Diet	X	X	X	X	X	X
Fluoride	X	X	X	X	X	X
Dental attendance			X	X	X	X
*Clinical*						
Oral hygiene	X	X	X	X	X	X
Past caries	X	X	X	X	X	X
White spot lesions				X	X	X
Enamel defects						X
Dental appliance			X	X	X	X
Systemic health	X	X	X	X	X	X
Medication				X	X	
*Salivary and microbiological*						
Saliva flow rate		X	X	X	X	X
Saliva buffering capacity		X				
Mutans streptococci	X	X	X		X	X
Lactobacilli	X	X	X		X	

*NusCra* National University of Singapore Caries Risk Assessment, *CAMBRA* Caries Management By Risk Assessment, *ADA* caries risk assessment by American Dental Association, *CAT* America Academy of Pediatric Dentistry’s Caries Assessment Tool

Nowadays, no systematic reviews are available on the performances of standardized models. Recent reviews have attempted to assess the validity of different caries risk assessment models/factors [3, 10, 13]. Two reviews combined single clinical parameters and standardized caries risk assessment models [3, 13]. The only externally validated model was the Cariogram [13]; the accuracy of the standardized model was found to be limited in pre-school children, based on two papers [15, 16]. The search literature contained a time frame from 1966 to 2006 with a refresh in 2011, so the most recent papers were not included in the review. Otherwise, Tellez et al. [3] aimed to appraise the evidence in caries prediction of two standardized CRA models, Cariogram, and Caries Management by Risk Assessment (CAMBRA), and two guidelines of the American Dental Association (ADA) and the American Academy of Pediatric Dentistry (AAPD), taking into account six longitudinal studies. In this review, the literature search was also stopped in 2011. Senneby et al. [13] evaluated the association between previous caries experience, microbiological tests, buffering capacity, salivary flow rate, oral hygiene, dietary habits, socio-demographic variables and the future caries lesion development. The evidence was considered of low quality and was lacking in regards to the studied methods. The literature search was stopped in January 2015.

Starting from these premises, this review aimed to evaluate the current literature on standardized CRA models, verifying whether the risk level measured using different tools is associated with the actual caries status and/or the future caries increment.

## Methods

This systematic review was conducted and reported following the Preferred Reporting Items for Systematic Reviews and Meta-analyses (PRISMA Statement) checklist.

### Protocol and registration

The review method and planning were registered at Prospero (PROSPERO 2016:CRD42016038590).

### Eligibility criteria

Randomized controlled trial (RCT), cross-sectional studies, cohort studies, comparative studies, validation studies and evaluation studies, reporting CRA using standardized models in patients of any age related to caries data recorded by Decayed, Missing, Filled Tooth/Surface (DMFT/S) or the International Caries Detection and Assessment System (ICDAS) indices were included. Only papers in English published from the 1st of January 2000 to the 31st of December 2017 were collected. This time frame was chosen since no standardized CRA tools were studied before the year 2000 as emerged from a first evaluation made by two authors (GC and MGC).

### Information sources and search strategy

Three different electronic databases were searched: PubMed, Scopus® and Embase®. Two search strategies were used; the first included a combination of MeSH terms and key words: caries risk assessment, caries risk assessment models, caries risk assessment tools, caries risk epidemiology, caries risk profile, Cariogram, CAMBRA, PreViser, NUS-CRA, ADA caries risk assessment, CAT caries risk assessment, AAPD caries risk assessment and dental caries susceptibility. The second strategy included the search string “((dentistry) OR (dental caries) OR (caries)) AND ((caries risk assessment) OR (Cariogram) OR (CAMBRA) OR (AAPD) OR (CAT) OR (ADA) OR (nuscra) OR (NUS-CRA) OR (PreViser)) AND ((cross-sectional studies) OR (cohort analysis risk) OR (cohort studies) OR (clinical trial) OR (clinical study) OR (controlled clinical trial) OR (observational study))”.

### Study selection

Repeated papers were deleted after comparing the results from the two different search strategies using the three databases. Two authors (GB and MGC) independently examined all the abstracts of the papers (see Additional file 1 for the whole list). All the papers meeting the inclusion criteria were obtained in the full-text format. The two authors independently assessed the papers to establish whether each paper should or should not be included in the systematic review (see Additional file 2 for the list of the papers excluded at this stage).

### Data collection

Data collection was carried out using an ad hoc designed data extraction form without masking journal title or authors. Data were extracted by two authors (MGC, GC) independently. For each paper the following data were searched and recorded when available: a) the year of publication and duration of the study; b) details of the participants including sample size at baseline, age and country of origin; c) caries data including actual caries status, caries experience and caries increment measured through DMFT/S or dmft/s or ICDAS; d) Caries risk assessment including standardized model used and categorization of the risk levels; e) sensibility and specificity of the CRA model.

Row data were requested to authors of longitudinal studies to perform data synthesis and analysis.

### Assessment of risk of bias and risk of bias across studies

The risk of bias assessment was conducted by two authors (GC and MGC). The methodological quality of the included studies was scored according to the customized quality assessment tool developed by the National Heart, Lung, and Blood Institute and Research Triangle Institute International for Observational Cohort and Cross-Sectional Studies since, as reported in the result section no RCTs were obtain after studies’ selection [17] (see Additional file 3 for quality assessment of included studies). Disagreements between authors were resolved by discussion. Where this was not possible, other authors were consulted (PL).

### Synthesis of the results

To facilitate a comparison of the results from different studies, the caries values were organized in two-by-two tables. Based on these tables, sensitivity and specificity were calculated, along with the corresponding 95% confidence intervals.

## Results

This review provides a concise description of the findings of the included papers, structured around the association between the standardized CRA models, performed on children and/or adults, and the actual or the predicted caries status.

**Fig. 1:**
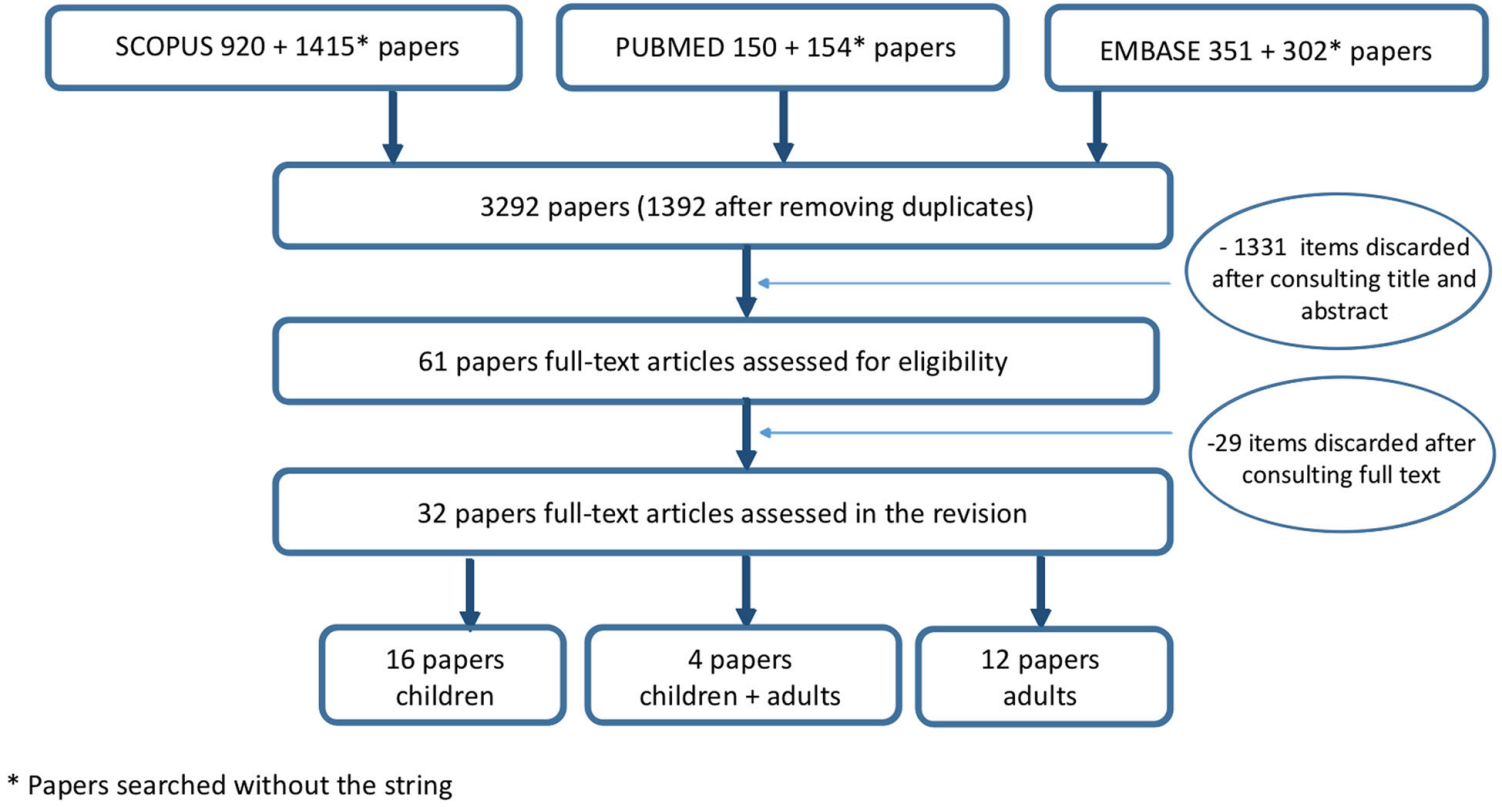
Flow chart of the study

The search identified 3326 papers; after removing duplicates, 1934 papers were selected and, after reviewing titles, abstracts and texts, 32 papers were finally included: 16 on children, 12 on adults and 4 on both, 3 of which considered children and adults as a single sample and one as two different samples (Fig. 1). In order to record caries status (experience/prevalence/incidence), 9 papers used DMFT index or sub-components, 13 papers used DMFS index or sub-components and only 1 used the ICDAS. Four papers focused on primary teeth, 24 on permanent teeth and 4 on both dentitions. The majority of papers (*n* = 31) estimated the caries risk using the Cariogram as a single model or in comparison with other models. No RCTs were included in the systematic review. All the considered longitudinal papers were comparative studies or validation studies or retrospective cohort studies or, finally, evaluation studies. All the included papers along with the quality assessment grade are reported in Table 2. Nineteen papers were classified as being of good quality, 9 papers of fair quality and only 4 of low quality.

**Table 2:** Papers included. Association between standardized CRA and actual caries status and/or caries prediction

Authors (year)	Outcome	Subjects	Indices	Caries risk model	Statistical significance	Quality assessment
Children/Adolescents						
Gao^b^, (2015) [8]	CI	544	dmft	Full Cariogram, CAT, CAMBRA, NUS-CRA	**+**	Good
Sundel, (2015) [21]	ACS	133	dmfs/DMFS	Full Cariogram	+	Good
Cabral, (2014) [22]	ACS	150	dmft/DMFT	Form based on Cariogram 7 factors	++	Poor
Kemparaj, (2014) [37]	CI	200	DMFT/S	Full Cariogram	+	Poor
Gao^b^, (2013) [36]	CI	544	dmft	Full Cariogram, CAT, CAMBRA, NUS-CRA	+	Good
Zukanovich, (2013) [41]	CI	109	DMFS/DMFT	Full Cariogram, PreViser, CAT	+	Fair
Campus, (2012) [35]	CI	957	DFS	Cariogram 7 factors	+	Good
Hebbal, (2012) [23]	ACS	100	DMFT	Full Cariogram	++	Poor
Kavvadia, (2012) [24]	ACS	814	dmft	Full Cariogram	+	Fair
Gao, (2010) [16]	CI	1576	dmft	Full Cariogram	^**e**^	Good
Petersson^c^,(2010b) [39]	CI	392	DMFS	Full Cariogram, Cariogram 6 factors	+	Good
Petersson, (2010a) [40]	CI	392	DMFS	Full Cariogram	+	Good
Campus, (2009) [25]	ACS	957	dmfs/DMFS	Cariogram 7 factors	++	Good
Holgerson, (2009) [15]	CI	125	dmfs/DMFS	Full Cariogram	+	Fair
Twetman, (2005) [33]	CI	64	DFS	Full Cariogram	++	Good
Petersson^a,b^, (2004) [34]	CI	446	DFS	Full Cariogram	++	Good
Petersson^b^, (2002) [32]	CI	446	DMFT/S	Full Cariogram	+	Good
Adults						
Petersson, (2015) [44]	CI	1295	DFT/DFTS	Full Cariogram	++	Good
Carta, (2015) [31]	ACS	480	ICDAS	Full Cariogram	++	Good
Chaffee, (2015) [45]	CI	4468	DFS	CAMBRA	+	Good
Chang (2014) [30]	CI	110	DMFT/S	Cariogram 7 factors	+	Good
Chang and Kim, (2014) [42]	ACS	102	DMFT	Full Cariogram	+	Good
Lee, (2013) [29]	ACS	80	DMFT	Full Cariogram, Cariogram 7/8 factors	+	Fair
Petersson, (2013) [7]	ACS	1295	DFT/S	Cariogram 8 factors	++	Good
Celik, (2012) [43]	CI	100	DMFT/S	Full Cariogram	+	Fair
Peker, (2012) [27]	ACS	90	DMFT/S	Full Cariogram	+	Fair
Sonbul, (2008) [28]	ACS	175	DMFS	Full Cariogram	+	Good
Ruiz Miravet, (2007) [26]	ACS	48	DMFT/S	Full Cariogram	++	Poor
Petersson^a,d^, (2004) [34]	CI	208	DFS	Full Cariogram	++	Fair
Petersson^d^, (2003) [1]	CI	208	DMFS /DFS/DFRS	Full Cariogram	++	Good
Both Children/Adults						
Giacaman, (2013) [20]	ACS	180	DMFT	Cariogram 7 factors	–	Poor
Almosa, (2012) [18]	ACS	89	DMFS	Full Cariogram	++	Fair
Al Mulla, (2009) [19]	ACS	100	DFS	Full Cariogram	++	Fair

*ACS* actual caries status, *CI* Caries Increment. Subjects: number of subjects at baseline

Statistical significance: **-** = *p* > 0.05 **+** =*p* < 0.05; **++** = *p* ≤ 0.01

^a^Petersson, (2004) reported in both children and adults and describes data in two different samples

^b^Gao, (2013) and (2015), and Petersson, (2002) and (2004) respectively reported data for the same sample of children

^c^Petersson, (2010a) and (2010b) reported data for the same sample of children

^d^Petersson, (2003) and (2004) reported data for the same sample of elderly people

^e^Data not obtainable from the paper

### Association between caries risk level and actual caries status in children

Two papers [18, 19] evaluated the association between caries prevalence (DMFS) and Cariogram 9 factors (hereinafter named Full Cariogram) in orthodontic patients. The low caries group at baseline displayed a statistically significant difference regarding caries increment and Cariogram level. Neither DMFT nor the number of caries lesions differed significantly in the Cariogram’s risk categories (7 factors) in a sample of Chilean subjects [20]. Children with a cleft lip and/or palate and non-cleft controls classified in the Cariogram high-risk category had a higher caries experience [21]. A significant linear regression between mean dmft and caries risk categories assessed according to a form based on the Cariogram was found in children from low-income families (*p* < 0.01) [22]. A statistically significant association between caries experience and Cariogram categories was found (*p* < 0.01) in Indian children [23]. Caries experience and the presence of white spot lesions were statistically significantly associated with Cariogram categories in Greek pre-school children (*p* < 0.01) [24]. A significant linear trend between the five Cariogram categories and dmfs/DMFS scores was observed (*p* < 0.01) in Italian children [25].

### Association between caries risk level and actual caries status in adults

Several papers focused on young adults and all of them reported an association between Cariogram categories and caries prevalence/experience/severity [7, 26, 27]. In Saudi Arabia [28], the mean caries prevalence in the high-risk group differed significantly from that recorded in the low-risk group (*p* < 0.05). A caries profile obtained from the Cariogram, including 7 and 8 factors, was compared to the Full Cariogram and correlated to caries experience: all models measured statistically significant associated risk levels to the caries experience [29]. The chance of avoiding caries was statistically significantly associated (*p* < 0.01) to the caries experience in a group of Korean adults [30]. Caries at ICDAS levels 5–6 and the presence of more than five missing teeth were statistically significant associated to the Cariogram scores (OR = 2.36, 95%CI = 1.83–3.03 and OR = 1.43, 95%CI = 1.13–1.82 respectively) in Italian adults [31].

### Association between caries risk level and caries increment in children and adults

A total of 17 longitudinal papers investigated the validity of standardized CRA models to predict new caries lesions (Table 3). Twelve papers regarding children were included [15, 16, 32–41], nine of which used the Cariogram model and three compared different CRA models, including the Cariogram [36, 38, 41]. Six papers regarding adults were included, five of which used the Cariogram [1, 34, 42–44] and one the CAMBRA model [45].

**Table 3:** Association between caries increment and caries risk model categories in longitudinal papers

Authors (year)	Age	Study time (years)	Subjects	Caries increments	Range Mean (Standard Deviation)				
				***Cariogram***	**0–20**	**21–40**	**41–60**	**61–80**	**81–100**
Gao (2013) [36]	C	1	485	dmft	2.67 (2.96)	2.02 (1.71)	1.56 (1.63)	0.77 (1.21)	0.34 (0.88)
Kemparaj (2014) [37]	C	2	200	DMFT	0.54 (1.2)	0.43 (1.32)	0.39 (1.04)	0.34 (0.80)	0.06 (0.09)
				DMFS	0.79 (1.73)	0.73 (1.55)	0.48 (1.72)	0.39 (1.20)	0.09 (1.12)
Celik (2012) [43]	A	2	100	DMFT	1.23 (0.86)	0.65 (0.81)	0.39 (1.02)	0.08 (0.28)	0 (0)
				DMFS	1.23 (0.86)	0.9 (0.97)	0.48 (1.6)	0.08 (0.28)	0 (0)
Petersson (2002) [32]	C	2	392	DMFT	1.67 (1.44)	1.46 (2.20)	1.07 (1.36)	0.42 (0.90)	0.23 (0.61)
				DMFS	2.58 (1.83)	2.62 (4.11)	1.47 (1.81)	0.53 (1.24)	0.27 (0.70)
Petersson (2015) [44]	A	3	982	DFT	1.00 (1.40)	0.84 (0.95)	0.82 (1.18)	0.53 (1.07)	0.24 (0.58)
Petersson (2010a) [40]	C	2	392	DMFS	3.00 (^a^)	2.70 (^a^)	1.50 (^a^)	0.50 (^a^)	0.20 (^a^)
				DFS	1.99 (3.00)	1.7 (1.76)	1.59 (2.55)	0.85 (1.91)	0.29 (0.89)
Petersson (2004)^b^ [34]	C	2	392	DFS	1.30 (^a^)	1.30 (^a^)	0.70 (^a^)	0.30 (^a^)	0.10 (^a^)
	A	5	148	DFS	1.90 (^a^)	1.00 (^a^)	1.20 (^a^)	0.40 (^a^)	0 (^a^)
Campus (2012) [35]	C	2	861	DS	1.20 (^a^)	1.20 (^a^)	0.10 (^a^)	0.20 (^a^)	0.10 (^a^)
				***Cariogram***	**0–20**	**21–40**	**41–60**	**61–100**	
Chang and Kim (2014) [42]	C	1.3	64	DMFT	2.97 (5.2)	1.28 (1.5)	1.36 (2.2)	0.44 (0.7)	
				DMFS	5.81 (11.97)	1.28 (1.5)	3.27 (6.8)	0.44 (0.7)	
Petersson (2003) [1]	A	5		DMFS	16.21 (15.97)	7.36 (9.34)	7.96 (9.52)	5.23 (6.97)	
				***Cariogram***	**0–25**	**26–50**	**51–75**	**76–100**	
Twetman (2005) [33]	C	3	64	DFS	8 (10.8)	3.4 (2.6)	2.6 (3.7)	0 (0)	
				***Cariogram***	**0–20**		**21–80**	**81–100**	
Zukanovic (2013) [41]	C	3	70	DMFT	1.80 (1.79)		2.40 (2.36)	1.77 (1.88)	
				DMFS	5.00 (7.07)		4.71 (4.34)	2.54 (2.44)	
				***Cariogram***	**0–40**			**41–100**	
Holgerson (2009) [15]	C	5	125	dmfs/DMFS	2.40 (3.2)			0.10 (0.4)	
				***Cambra***	**High**		**Moderate**	**Low**	
Gao (2013) [36]	C	1	485	dmft	1.24 (1.58)		0.27 (0.68)	0.20 (0.76)	
Chaffee (2015) [45]	A	1.5	4468	DFT	1.74 (^a^)		1.16 (^a^)	1.01 (^a^)	
				***CAT***	**High**		**Moderate**	**Low**	
Gao (2013) [36]	C	1	485	dmft	0.79 (1.31)		0.08 (0.28)	0 (0)	
Zukanovic (2013) [41]	C	3	70	DMFT	2.19 (2.33)		2.60 (1.82)	2.38 (1.92)	
				DMFS	4.54 (4.41)		3.80 (5.81)	3.13 (2.53)	
				***NUS-CRA***	**Very High**	**High**	**Moderate**	**Low**	**Very Low**
Gao (2013) [36]	C	1	485	dmft	2.18 (1.87)	2.10 (1.63)	1.26 (1.38)	0.85 (1.11)	0.17 (0.69)
				***PreViser***	**High**		**Moderate**	**Low**	
Zukanovic (2013) [41]	C	3	70	DMFT	2.35 (2.27)		1.92 (2.18)	2.18 (2.32)	
				DMFS	5.04 (4.75)		3.08 (2.87)	2.82 (3.19)	

*A* Adults, *C* Children

(^a^) indicates that Standard Deviation data were not described in the paper. The decimal places reported are those reported in each paper

Petersson, (2004)^b^ reports the increment for year of observation. Holgerson, (2009) and Petersson, (2010b) were excluded from the table since as no mean data for caries were present. Gao (2015) was excluded from the table as the data are the same as those reported for Gao, (2013)

In a two-year prospective study [32] subjects in the highest risk group developed a mean of about 10 times more caries lesions (DMFS) than the lowest risk group. The same authors compared [34] data from the previous study with those recorded in a group of adults/elderly people, showing a higher mean of caries increment per year for high-risk groups. The caries increment in children affected by Type 1 diabetes mellitus (ΔDMFS) was about eight times higher in the Cariogram highest risk category [33]. After five years from baseline, children classified at high risk (Full Cariogram) developed about four times more caries lesions [15] (study not included in Table 3). Five times more caries lesions were found in schoolchildren assessed by the Full Cariogram as running the highest risk compared with those with the lowest risk [40]. On the same sample, two different Cariogram models with and without saliva factors were tested. Both models revealed a statistically significant relationship with caries development (*p* < 0.05) at the two-year follow-up [39]. A prospective study (not included in Table 3) was conducted on preschool children with different risk assessment models, including the Full Cariogram [16]. One year after baseline the model showed a sensitivity/specificity lower values than the biopsychosocial models proposed by the authors. In Italian schoolchildren, the caries risk was assessed (7-factor Cariogram) and 2 years later the children classified as high risk developed caries lesions about as twice as much as those developed by children classified as low risk [35]. Full Cariogram, Previser and the Caries-risk Assessment Tool (CAT) were compared in children [41]. At the follow-up examination (3 years), only the Cariogram model successfully predicted new caries lesions. Full Cariogram, NUS-CRA, CAT and CAMBRA were assessed on preschool children [36]. After 1 year, using CAT and CAMBRA, the majority of children were considered to be at high risk, while, using the Full Cariogram and the National University of Singapore Caries Risk Assessment (NUS-CRA), almost 2/3 of the children were defined as very low or low risk. The CRA was evaluated in a sample of 12-year-old children using the Full Cariogram and dividing them into five groups of risk [37]. Two years later, children classified as very high risk at baseline developed about thirty times more caries lesions compared to children classified as very low risk. The same sample of three-year-old children from a previous study [36] was re-evaluated 18 months from baseline (*n* = 462) using the same risk assessment model; a gradient in caries increment from lower to higher risk groups was found using all programs [38].

Full Cariogram was evaluated in elderly people and, after 5 years, subjects with the highest risk profile had about three times more caries lesions compared to the lowest risk group [1]. Full Cariogram was assessed in two samples of young adults [43, 44]; after 2 years, subjects classified at very high risk at baseline developed caries lesions about as twice as much as those classified at very low risk; at the 3 year follow-up of the second sample [44], subjects with the highest risk profile had about seven times more caries lesions compared to those in the lowest risk group. The CAMBRA model was used to split a sample of young adults into four risk groups [45]; caries increment was more than three times higher in subjects classified as high risk than those classified at low risk.

Few of the included papers report data allowing the authors to calculate sensibility and specificity of the CRA models [15, 16, 32, 33, 35, 36, 44]. In Table 4 the available data for the Cariogram model are displayed. Sensibility values ranged from low (41.0) [32] to fairly low (52.0) [35], while specificity values were quite high, ranging from 71.0 [33] to 88.0 [15]. Moreover, wide Confidences Intervals are reported for both parameters, indicating that the reliability of the model differs in the different caries risk levels.

**Table 4:** Sensitivity and specificity of the Cariogram model in children and adults

Authors (year)	Number of factors	Sample n	Age at baseline (years)	Sensibility % (_95%_Confidence Interval)	Specificity % (_95%_Confidence Interval)
Children					
Gao (2013) [36]	Full	485	3	66.4^a^	78.5^a^
Campus (2012) [35]	7	861	7–9	52.0 (18.6–94.6)	79.5 (99.2–54.7)
Gao (2010) [16]	Full	1782	3–6	70.5^a^	65.8^a^
Holgerson^b^ (2009) [15]	Full	66^b^	2	46.0 (31.0–62.0)	88.0 (71.0–104.0)
Twetman (2005) [33]	Full	64	8–16	75.0^a^	71.0^a^
Petersson (2002) [32]	Full	392	10–11	41.0 (9.0–73.0)	79.8 (99.6–60.0)
Adults					
Petersson (2015) [44]	Full	1295	19	47.0 (11.9–89.2)	72.5 (33.5–94.8)

^a^Range not available

^b^Control group only

In brief, the results of the present review show: all the included papers on children showed a statistically significant association between the risk levels and the actual caries status and/or the future increment. More than half of these papers, including the Cariogram model, were classified as being of good quality. The same positive association between the risk levels and the actual caries status and/or the future increment was reported in the included papers on adults. More than half of the papers were classified as being of good quality and all except one used the Cariogram. Three of four papers comprising children and adults found a positive association between the risk levels and the actual caries status and/or the future increment.

## Discussion

Determining the validity of different caries risk assessment models to fit the actual caries status, analyzing cross-sectional papers, and to predict new caries lesions in the near future, analyzing longitudinal papers, was the aim of this systematic review. The CRA models that were examined were the reasoning-based (CAT, CAMBRA and ADA model) and algorithm-driven (Cariogram, NUS-CRA and PreViser).

The findings described enable to draw some conclusions.

All papers involving children [15, 16, 21–25, 32–41] assessed a statistically significant association between the risk level measured by the CRA model and the actual caries status or the caries increment in a follow-up examination. Eleven papers [16, 21, 25, 32–36, 38–40] of seventeen were classified as being of good quality, and all of them used the Cariogram as sole model or in conjunction with other models. Sensibility and specificity of the Cariogram model were evaluated in six papers [15, 16, 32, 33, 35, 36] and data showed that the model is not accurate in predicting caries lesion development. Furthermore, the validity of the Cariogram to evaluate the caries risk might be flawed: four papers [32, 34, 39, 40] involved the same population.

Papers carried out on adult populations [1, 7, 26–31, 34, 42–45] showed a positive association between the CRA model and caries data. Eight papers [1, 7, 28, 30, 31, 42, 44, 45] of thirteen were classified as being of good quality and all except one [45] used the Cariogram model. Sensibility and specificity of the Cariogram model were reported in one paper [44]; data confirm the low accuracy of the model. Within the three papers [18–20] involving both children and adults regarded as a single sample, two found a positive association between risk level and caries status and one [20] failed to find such an association. This last paper was evaluated as being of poor quality.

Different Cariogram models using from nine to seven factors were used. The excluded factors were salivary parameters, namely mutans streptococci, lactobacilli, salivary secretion rate and buffer capacity. Reduced Cariogram versions were statistically significant associated to caries data, in both cross-sectional and longitudinal papers [7, 21, 29, 30, 35], except for one paper [20].

Only three papers, two of good quality (reporting data on the same sample) [36, 38] and one fair, compared different risk models [41]: the Full Cariogram, CAT, CAMBRA and NUS-CRA were compared in two and the Full Cariogram, PreViser and CAT in the third one. The results showed that different CRA models assessed the risk differently, but, due to the small amount of available data, it is not possible to draw clear conclusions about the most effective method for predicting caries lesions.

The main limitation of this review is that the included papers do not form a homogeneous group and original databases are not available making it impossible to perform a meta-analysis. Different study populations (adults or children), different versions of the same standardized CRA model (Cariogram from seven to nine parameters), different indices used to measure carious lesions (dmfs/t, DMFS/T, DFS/T, DS, ICDAS) make the comparison of papers questionable and hamper the synthesis of results. This limitation cannot be overcome until papers with a standardized study design, outcome measurements and reporting of data will be carried out.

At present, only the Cariogram was used in papers of good quality to assess its efficacy in predicting caries development, while, for the other standardized CRA models, the lack of papers does not make it possible to draw conclusions on their effectiveness.

## Conclusions

The evidence relating to the quality of existing CRA models in assessing and predicting caries lesions is limited; even if Cariogram was used in few studies of good quality carried out in children and adults, no sufficient evidence is available to affirm that the method is effective in caries assessment and prediction. The Full Cariogram and reduced versions, eight or seven factors, appear to produce similar results. Although other CRA models, such as CAT, CAMBRA, NUS-CRA and PreViser, might be effective in clinical settings, the scientific evidence to date is limited.

## Additional files

Additional file 1: List of papers excluded in the first selection (XLSX 54 kb)

Additional file 2: List of papers excluded in the second selection (XLSX 11 kb)

Additional file 3: Quality assessment (XLSX 13 kb)

## Abbreviations

AAPD: American academy of pediatric dentistry
ADA: American dental association
CAMBRA: Caries management by risk assessment
CAT: Caries risk-assessment tool
CRA: Caries risk assessment
DMFT/S: Decayed missed filled tooth/surface
ICDAS: International caries detection and assessment system
NUS-CRA: National university of singapore- caries risk assessment
PRISMA: Preferred reporting items for systematic reviews and meta-analyses
RCT: Randomized clinical trial
